# Diverse environmental cues drive the size of reproductive aggregation in a rheophilic fish

**DOI:** 10.1186/s40462-023-00379-0

**Published:** 2023-03-22

**Authors:** Marek Šmejkal, Daniel Bartoň, Petr Blabolil, Tomáš Kolařík, Jan Kubečka, Zuzana Sajdlová, Allan T. Souza, Marek Brabec

**Affiliations:** 1 Institute of Hydrobiology, Biology Centre of the Czech Academy of Sciences, České Budějovice, Czech Republic; 2 Institute for Atmospheric and Earth System Research INAR, Forest Sciences, Faculty of Agriculture and Forestry, University of Helsinki, Helsinki, Finland; 3 Institute of Computer Science, Czech Academy of Sciences, Prague, Czech Republic

**Keywords:** Weather, Reproductive behaviour, Long-term monitoring, Fish movement, Migration, Phenology

## Abstract

**Background:**

Animal migrations are periodic and relatively predictable events, and their precise timing is essential to the reproductive success. Despite large scientific effort in monitoring animal reproductive phenology, identification of complex environmental cues that determine the timing of reproductive migrations and temporal changes in the size of reproductive aggregations in relation to environmental variables is relatively rare in the current scientific literature.

**Methods:**

We tagged and tracked 1702 individuals of asp (*Leuciscus aspius*), a large minnow species, and monitored with a resolution of one hour the size of their reproductive aggregations (counts of sexes present at the breeding grounds standardized by the sum of individuals in the season) over seven breeding seasons using passive integrated transponder tag systems. We examined the size of reproductive aggregations in relation to environmental cues of day number within a reproductive season (intra-year seasonality), water temperature, discharge, hour in a day (intra-day pattern), temperature difference between water and air, precipitation, atmospheric pressure, wind speed and lunar phase. A generalized additive model integrating evidence from seven breeding seasons and providing typical dynamics of reproductive aggregations was constructed.

**Results:**

We demonstrated that all environmental cues considered contributed to the changes in the size of reproductive aggregations during breeding season, and that some effects varied during breeding season. Our model explained approximately 50% of the variability in the data and the effects were sex-dependent (models of the same structure were fitted to each sex separately, so that we effectively stratified on sex). The size of reproductive aggregations increased unimodally in response to day in season, correlated positively with water temperature and wind speed, was highest before and after the full moon, and highest at night (interacting with day in a season). Males responded negatively and females positively to increase in atmospheric pressure.

**Conclusion:**

The data demonstrate complex utilization of available environmental cues to time reproductive aggregations in freshwater fish and their interactions during the reproductive season. The study highlights the need to acquire diverse data sets consisting of many environmental cues to achieve high accuracy of interpretation of reproductive timing.

**Graphical abstract:**

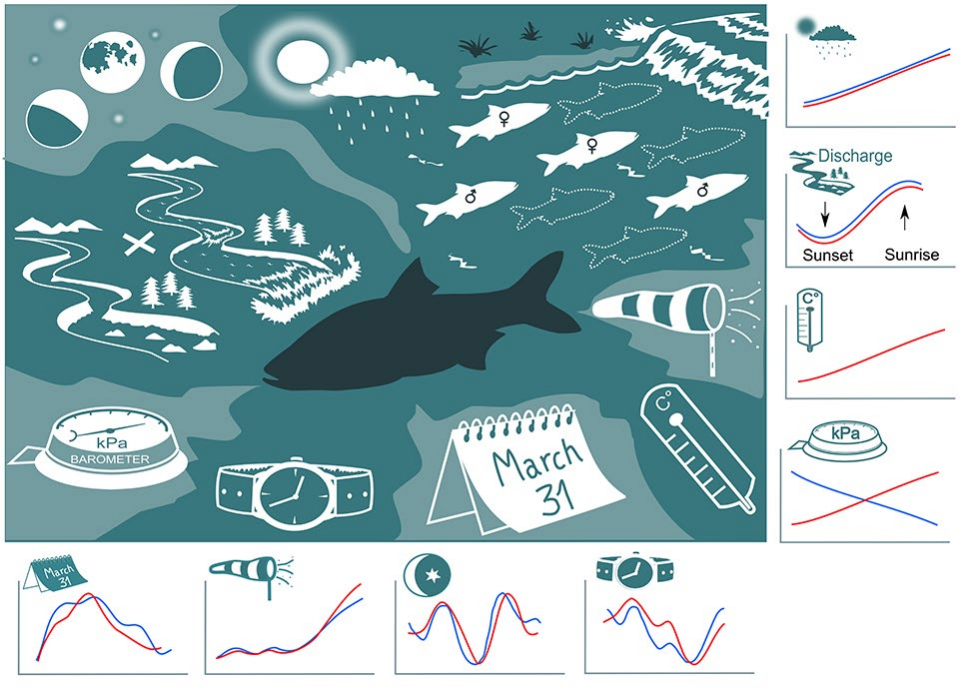

## Introduction

Migrations of entire animal populations or their parts have evolved independently in many animal taxa [1–3]. The reasons why animals move from one habitat to another are usually threefold: improved food acquisition through habitat change [4], predation avoidance strategy [5], or reproduction [6]. For the latter in particular, the phenology of migrations and the onset of reproduction presents a complex challenge for both individuals and populations, as the precise timing of reproduction is paramount to reproductive success due to the need to match offspring feeding demands with high food availability [7, 8]. Both too early or too late migration may result in lower fitness, higher energy expenditures and risk of adult mortality [9–12], placing strong selection pressure on the migration phenology. Therefore, animals must acquire reliable environmental information about future food availability and suitability of environmental conditions for their offspring [13, 14].

With their diverse migration strategies, fishes are one of the most studied animal groups in the field of migration [15]. Nevertheless, their migration trends are often not well understood, and much of the migration variance is not properly explained by the few environmental variables typically used in models [16]. Different fish species have been found to use different environmental cues to time their reproductive migration, such as photoperiod [17], temperature [18], discharge [19], hour in a day [20], lunar cycle [21], atmospheric pressure [22], or precipitation [23]. In fishes, especially the first two environmental cues are believed to be fundamentally important for gonadal maturation and reproductive migrations [24, 25]. Together with photoperiod, which regulates physiological processes, water temperature appears to be a determining cue that interacts with the photoperiod signal to synchronize the final stages of reproductive development with optimal environmental conditions [26]. In weak seasonal variation in photoperiod and temperature, such as the ones in tropical regions, environmental cues related to current weather conditions and tidal strength may be more relevant for the timing of the onset of migration or reproduction itself [23, 27]. In the case of nocturnal migrations aimed at avoiding the risk of predation on migrating adults, low light levels associated with the phase of the moon may also be an important cue for migration [28].

Timing of reproductive migrations have mostly been studied in terms of environmental cues that trigger migration from feeding to breeding grounds [23]. However, upon arrival near breeding grounds, some species gather at staging grounds days to weeks before the onset of reproduction, waiting for specific environmental cues that determine the final timing for the onset of reproductive effort [29, 30]. In the final stages of reproduction, animals depart from staging grounds to breeding grounds, often multiple times per season and possibly with distinct sex-specific patterns [29, 31]. This leads to fluctuations in the size of reproductive aggregations, in which reproducing individuals on the reproductive grounds are periodically assembling and disassembling, usually with daily periodicity and in varying numbers [27, 32, 33].

In many animal taxa, males migrate to reproductive grounds earlier than females, leading to a phenomenon known as protandry [28, 34]. In territorial males, early arrival enables the acquisition of higher value territory and thus better chances of high fitness in the reproductive season [35, 36]. In non-territorial species, the advantage comes from the higher number of females encountered compared to the late arrival strategy [37]. Individuals and sexes with different arrival strategies may also evaluate the same environmental cues differently, resulting in their different arrival to and departure from breeding grounds [38, 39].

In this study, we investigated the contribution of nine environmental cues to the size of reproductive aggregation by tagging 1702 asp (*Leuciscus aspius*) and tracking their presence on the breeding grounds over the course of seven consecutive reproductive seasons. Specifically, we examined the role of day of a season, water temperature, discharge, hour in a day, difference between water and air temperature, precipitation, atmospheric pressure, wind speed and lunar phase on the size of reproductive aggregation. Our models were stratified by sex since males and females exhibit distinct migration behaviours, with males arriving on spawning grounds an average of 5 days earlier than females [38], and thus likely evaluate environmental cues differently.

## Materials and methods

### Studied species

**Fig. 1:**
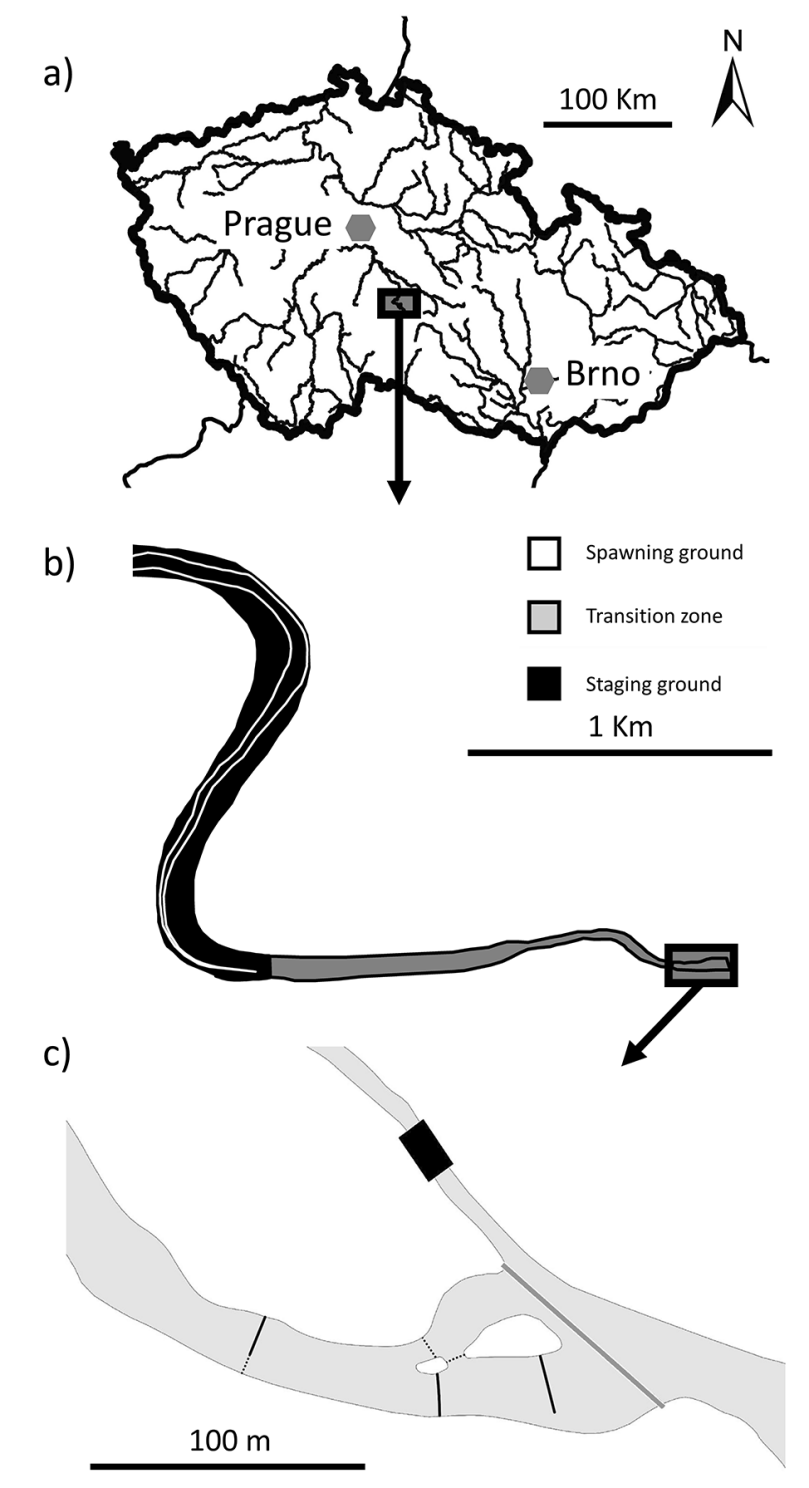
Map of (a) position of studied location in the Czech Republic, (b) spawning and breeding ground and (c) detail of breeding grounds with the antenna deployment setup. White line depicts the occurrence of depth 3 m or lower. Antenna deployment setup: placement of antenna systems is indicated by solid black lines. Nets guiding asps into passing through the antennas are indicated by dotted lines. The migration further upstream (right side direction) is restricted by a weir (solid grey line) and reproduction occurs in the most fluvial part between the weir and the second antenna. We used QGIS software for the graphical visualization [82]

The asp is a European fish species of the Leuciscidae family that inhabits lakes and rivers and thrives in many man-made reservoirs. In early spring, the asp migrate to fast flowing rivers to breed [40]. It is an open substrate spawner, classified as lithophilous, with no parental care and adhesive eggs that hatch about 20 days after deposition, depending on water temperature in spring [41, 42]. The range of water temperature utilized for breeding is relatively broad and ranges between 5 and 14 °C [43]. Both sexes arrive and depart breeding grounds multiple times per reproductive season (Fig. 1), which leads to varying size of reproductive aggregation with mostly nocturnal abundance peaks and using staging grounds located 1–3 km downstream for rest [20]. Males arrive earlier and depart later than females to maximize their reproductive output, and their performance improves with age [29]. It is a relatively long-lived species with iteroparous reproduction and the maximum of six recorded spawning seasons for an individual (author´s unpublished data). The reproductive period is the only time when sex of asp can be visually determined with certainty based on morphological and physiological characteristics (see below in the section “Capture and tagging of fish”).

### Study site

The study was performed in the tributary of the Želivka Reservoir in the Czech Republic (49°57’83"N, 15°25’37"E). The dam construction generated a 39.1 km long artificial water body with an area of 1602 ha and a maximum water level of 381.7 m above sea. A weir located in the river just hundreds of m (depending on the reservoir water level) upstream of the reservoir blocks further upstream migration of asp and restricts its reproduction within the studied system to breeding grounds less than 100 m long (Fig. 1), that have been monitored by passive telemetry since 2015 [31].

### Capture and tagging of fish

Fish were captured at staging grounds using an electrofishing boat (electrofisher EL 65 II GL DC, Hans Grassel, Schönau am Königsee, Germany; 13 kW, 300/600 V, 5–10 A, pulsed DC; frequency: 70 × s^− 1^) during breeding seasons from 2014 to 2021. Fish were sampled at staging grounds (water depth: 0.5–5 m), with an approximately 6 m wide and 3 m deep energised field, to which fish are attracted and paralysed [44]. Once an individual was paralysed, the boat crew stopped the electric pulses, retrieved the fish from the water and placed it in an aerated tank filled with reservoir water. The fish were anaesthetized with MS-222, and their standard length (in mm), sex, and weight (in g) were recorded. Three main characteristics were used to distinguish the sex of the fish: release of milt, breeding tubercles and slender bodies in males, and no tubercles, robust bodies and possible release of eggs when light pressure was applied to the fish belly in females. A passive integrated transponder tag (PIT tag, Oregon RFID, Oregon, USA; half-duplex; length: 32 mm; diameter: 3.65 mm; weight: 0.8 g; ISO 11,784/11,785 compatible) was used to provide fish´s individual identification code and allow its subsequent recognition in monitored breeding grounds. A PIT tag was inserted into the body cavity after removing 3–4 scales and performing a 4–5 mm vertical incision 3 cm behind the pelvic fin. Loss of PIT tags from scar wounds is minimal in males (less than 1%), but females are approximately 11% likely to expel their markings during oviposition [45]. Tagged asp were released immediately after recovery from anaesthesia. Females averaged 492 ± 49 mm and males were of 467 ± 39 mm standard length. The percentage of tagged fish in total shoal size in given year represented 8–45% of migrating males and females depending on sex and year of monitoring (Table 1).

**Table 1:** Summary of tagging effort, percentage of tagged fish from previous years to fish caught by electrofishing, number of detected fish tagged in the previous season in each year, and monitoring periods (starting and ending date of sampling in given year)

	2014	2015	2016	2017	2018	2019	2020	2021
Tagged M	220	209	361	281	162	101	203	276
Tagged F	135	181	252	303	201	88	127	143
Tagged M %	-	13.6	18.7	29.9	44.9	42.6	25.5	15.9
Tagged F %	-	7.6	9.0	14.4	30.0	29.0	22.4	16.3
Det. M	-	100	169	322	418	456	338	290
Det. F	-	53	106	238	365	436	287	275
Start	-	25 − 03	16 − 03	20 − 03	20 − 03	20 − 03	18 − 03	18 − 03
End	-	21 − 04	22 − 04	21 − 04	25 − 04	20 − 04	27 − 04	06 − 05
No. of antennas	-	2	3	3	3	3	4	3

### Recording the size of reproductive aggregations

Tagged asp that migrated to breeding grounds were recorded using passive telemetry systems (LF HDX RFID readers, Oregon RFID, Oregon, USA). The electromagnetic field generated in the antenna loop energizes the PIT tag of a tagged individual located in the antenna field, whereupon the PIT tag emits an individual fish code that is recorded and stored in the antenna reader along with the date and time. Since the fish aggregate at the breeding ground and some PIT tagged fish may be missed due to interference with other PIT tags [46], we constructed several antennas each reproductive season (Table 1). Number of antennas installed was based on the breeding ground size (the length of fluvial river section) with 2–4 antennas in a reproductive season located approximately 50 m apart to avoid occurrence of noise in the antenna signal (Fig. 1; Table 1). The asp were guided by barriers to swim through the 10 m wide and 0.5–1 m high electromagnetic field [29]. The system scanning frequency was set to 10 energizing and reading cycles×s^− 1^, and the systems were tested daily (by manually inserting a PIT tag into antenna field) to ensure that the antennas were scanning their entire detection range. The time of the antennas was set and updated weekly to ensure the time in each system match. Antenna deployment and continuous monitoring in a given year began prior to the onset of asp breeding (no or few asp were detected during the first few days of monitoring) and continued until the most asp had disappeared from the breeding grounds.

### Abiotic data collection

Data on weather conditions based on the so-called meteorological re-analysis (complex retrospective data processing technique that essentially includes data from terrestrial and satellite measurements and numerical weather modelling outputs) [47]. Data were retrieved with a temporal resolution of 1-h and a spatial accuracy of 1 × 1° from the NASA – MERRA-2 project [48, 49] using the application programming interface (API) provided on the website. Specifically, air temperature in °C (2 m above ground), precipitation in mm×m^− 2^, atmospheric pressure and wind speed at 10 m above the ground were obtained for the dates of the sampling campaigns and selected for subsequent analysis.

The elevated air temperature (captured by variable differences between air and water temperature) was incorporated in the analyses since it can be indicative of fish basking in warm upper water layers [50] to accelerate gonad maturation. Precipitation, changes in atmospheric pressure and wind speed were involved in the model since these variables are indications of changing weather and thus potentially important parameters for reproductive timing. Water discharge data (m^3^ × s^− 1^) were obtained at 1-h resolution from the automated monitoring station 6.5 km upstream owned by the Vltava River Authority state enterprise (www.pvl.cz), and the time was adjusted for the delayed arrival of the discharge change at the research site. Temperature during the monitoring campaigns was measured with dataloggers (HOBO Pendant Temperature/Light 64 K Data Logger, Onset Computer Corporation, Bourne, Massachusetts, USA) placed in the breeding grounds at 0.5 m depth. Lunar phases for fish reproduction monitoring were obtained using the R package lunar [51].

### Data treatment and processing

The tagging procedure may affect fish behaviour in the first days after tagging [52], and newly tagged fish in the current year would bias the distribution of breeding fish due to the cumulative effect of tagging during season. Therefore, only fish tagged in previous breeding seasons were considered in the analysis of a given year.

Antennas were installed only at the breeding ground, so any individual recorded by one of the antennas was defined as migrant and counted as present. Due to possible PIT tag collisions and missed detections caused by more than one tagged fish in the antenna field [53], fish departure was defined as the absence of detection of a given individual for more than an hour after the last detection. A single detection of fish resulted in fish presence only in a given hour of detection. Due to fish reproductive activity and circular movement between antennas in search of mating partners [54], this system detects individual fish on average multiple times per hour (average detections of males per hour in 2015 and 2016: 27 and 55; female detections: 15 and 25, respectively) [31].

### Statistical analysis

Our statistical analysis was based on the following GAM (Generalized Additive Model) Gaussian model [55], which was stratified on sex (i.e. same form of the model was fitted separately for different sexes– as the AIC comparison of the sum of sex-stratified models was superior to the sex-interactive model, 34265.1 vs. 40830.7). We opted for aggregative response to avoid many issues with detailed modelling of the subtle correlation structure among different individuals. The model was constructed as follows:

$$\begin{array}{l}{Y_{ijk}} = {\beta _0} + {\beta _1} \cdot W{T_{ijk}} + {\beta _2} \cdot D{T_{ijk}} + {\beta _3} \cdot Precipitatio{n_{ijk}}\\+ {\beta _4} \cdot Pressur{e_{ijk}} + {s_{seas}}\left( i \right) + {s_{seas * hour}}\left( {i,\,j} \right) + {s_{hour}}\left( j \right) \cdot \\Discharg{e_{ijk}} + {s_{wind}}\left( {Win{d_{ijk}}} \right) + {s_{lunar}}\left( {L{P_{ijk}}} \right) + {\varepsilon _{ijk}}\end{array}$$

where:

- ${Y}_{ijk}$ was the size of reproductive aggregation (count of fish divided by sum of total individual hours) observed in year *k*, at the *i*-th relative day position (corresponding to the number of days since the first day of asp detection within the *k*-th season) in hour j.
- ${\beta }_{0},{\beta }_{1},{\beta }_{2},{\beta }_{3}$, ${\beta }_{4}$ were unknown constants (coefficients) to be inferred from the data.
- ${s}_{seas}$, ${s}_{seas*hour}$, ${s}_{hour}$, ${s}_{wind}$, ${s}_{lunar}$ were unknown smooth components implemented as complexity-penalized splines [56, 57] to be estimated from data.
- To reflect cyclic nature of the diurnal pattern, we implemented ${s}_{hour}$ as a cyclic cubic spline (using bs="cc” in mgcv’s gam). Furthermore, the cyclic cubic component was also used in the corresponding part of the interaction ${s}_{seas*hour}$, which was implemented as a tensor product spline (using bs = c(“cr”,“cc”)). Similarly, periodicity was enforced in the lunar phase component ${s}_{lunar}$. On the other hand, cyclicity was not enforced in the seas term (or the seas part of the tensor product spline), because we did not have year-round data (only relatively short parts of the year corresponding to breeding seasons).
- ${WT}_{ijk}$ was water temperature at the *j*-th hour of the *i*-th relative day position of season *k*.
- ${DT}_{ijk}$was the difference between ambient air and water temperature at the *j*-th hour of the *i*-th relative day position of season *k*.
- ${Precipitation}_{ijk}$was precipitation at the *j*-th hour of the *i*-th relative day position of season *k*.
- ${Pressure}_{ijk}$ was air pressure at the *j*-th hour of the *i*-th relative day position of season *k*.
- ${Wind}_{ijk}$ was wind speed at the *j*-th hour of the *i*-th relative day position of season *k*.
- ${LP}_{ik}$ was lunar phase of the *i*-th relative day position of season *k*.
- ${Discharge}_{ijk}$ was water discharge at the *j*-th hour of the *i*-th relative day position of season *k*.
- ${\epsilon }_{ijk}N\left(0,{\sigma }^{2}\right)$ was a random error

Therefore, our model had linear trends in water temperature, difference between ambient air and water temperature, precipitation, air pressure, then it contains smooth (potentially nonlinear) effects of wind speed, lunar phase and seasonal correction (where the seasonal effect was allowed to change with hour in a day, and the water discharge effect trend coefficient was also allowed to change with hour in a day). Formally, we had season*hour and hour*discharge parsimonious versions of interaction in the model. The hour*discharge component was formulated as the so called TVAR (time-varying effect) form [58].

All unknown model parameters (both functional and traditional) were estimated via maximization of (penalized) likelihood upon fixing the roughness penalties at their estimates from REML (restricted maximum likelihood). The computations were conducted in R [59] using the mgcv library [56].

We started with the default K value of GAM (which depends on the type of smoother) and inspected the output of gam.check along with the estimated smooth component. The K values were fairly close to 1 and the p values were far from significant, except for the lunar phase component. However, increasing the K value would cause the smoother to select details that were difficult to interpret and generalize. Therefore, we stuck with the default selection with the simple and unambiguous interpretation of the contrasting light and dark phases.

## Results

In the 2015–2021, the counts of tagged individuals contributing to reproductive aggregation estimates were ranging from 100 to 456 in males and 53 to 436 in females, respectively. The proportion of tagged individuals to the total number of migrating adults ranged from 13.6 to 44.9% in males and from 7.6 to 30.0% in females, respectively (Table 1). Environmental cues varied among seasons, with 2015 and 2021 being relatively coldest compared to 2017 and 2018 being relatively warmest (Table 2). Average discharge also varied among seasons with 2020 being relatively driest, and 2017 being relatively wettest season (Table 2). Atmospheric pressure and wind speed during breeding season were rather similar among seasons (Table 2).

**Table 2:** Variability of environmental cues (means and standard deviations) among studied breeding seasons 2015–2021. Day in a season, lunar phase, and hour in a day are not listed in this table due to their trivially predictable nature

Breeding season							
	2015	2016	2017	2018	2019	2020	2021
Water temp (°C)	6.9 ± 1.5	7.3 ± 2.0	8.3 ± 1.5	8.1 ± 4.1	7.6 ± 1.3	7.9 ± 2.8	7.0 ± 2.3
Temp. diff. (Air-water)	-1.3 ± 4.3	-0.5 ± 4.1	-1.5 ± 4.8	0.5 ± 4.3	-0.7 ± 4.8	-1.7 ± 4.9	-2.1 ± 4.6
Discharge (m^3^ × s^− 1^)	2.9 ± 2.3	4.4 ± 2.9	6.6 ± 4.5	3.7 ± 2.1	4.4 ± 2.2	2.1 ± 0.7	3.8 ± 1.5
Precipitation (mm/hour)	0.08 ± 0.20	0.05 ± 0.13	0.07 ± 0.20	0.03 ± 0.10	0.01 ± 0.03	0.02 ± 0.12	0.07 ± 0.25
Atm. pressure (kPa)	95.8 ± 0.9	95.5 ± 0.56	96.1 ± 0.5	95.4 ± 0.8	96.1 ± 0.9	96.2 ± 0.6	95.8 ± 0.6
Wind speed (m × s^− 1^)	5.3 ± 2.8	3.9 ± 1.8	4.4 ± 1.9	4.4 ± 2.0	4.2 ± 1.9	4.1 ± 2.0	4.4 ± 1.9

**Fig. 2:**
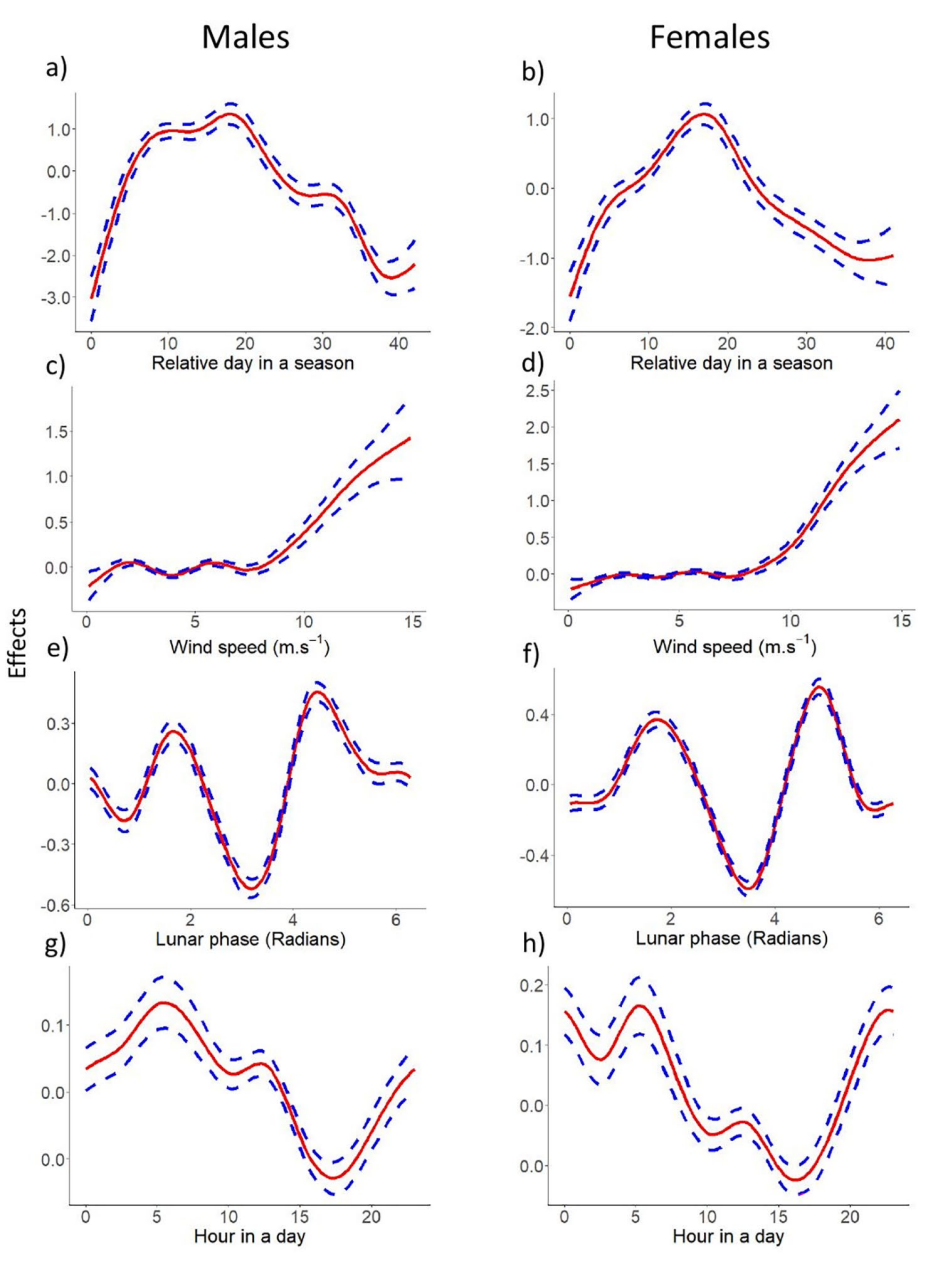
Effects of significant variables (relative day in the season, wind speed, lunar phase and interaction between discharge increase on hour in a day) in GAM models (i.e., estimated smooth effect of a given covariate) on the size of reproductive aggregations in females and males together with the (pointwise constructed) 95% confidence intervals. Red lines represent GAM model-estimated trends (on a logistic scale), and the dashed lines represent corresponding confidence intervals. Radians to moon phases: 1.5 = first quarter; 3 = full moon; 4.5 = last quarter; 6 = new moon

**Fig. 3:**
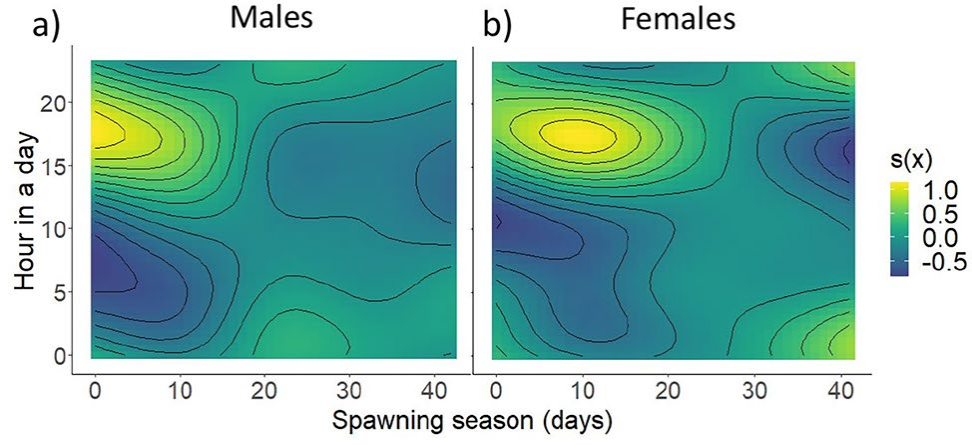
Dependence of breeding intensity between smoothing of breeding season and hour in a day resulting from GAM models. While the trend of reproductive aggregations in the beginning of breeding season is evening and nocturnal, this pattern disappears at later stages of breeding season

Both models were highly significant and explained around 50% data variability in the size of reproductive aggregations of both males and females. With the same mathematical form the adjusted R^2^ of the male model M1 (N = 5569) was 0.56 (56.3% explained deviance), whereas the female model M2 (N = 5537) had R^2^ of 0.488 (49.3% explained deviance). From the parametric coefficients included in the models, water temperature had no significant effect on the size of reproductive aggregations in males, while it had a significant effect on the size of reproductive aggregations in females (Table 3). The difference between air and water temperature was positively related to the size of reproductive aggregations in both males and females (Table 3). In addition, precipitation had a significant effect on the size of reproductive aggregations in both sexes, and decrease in atmospheric pressure had a significant positive effect on the size of reproductive aggregations in males, but a negative effect in females (Table 3). Regarding the seasonal aspect, the size of male reproductive aggregations peaked early in the season and remained high in the first half of the season and slowly decreased in the second half of the season. Female reproductive aggregations increased at a lower slope compared to males at the beginning of the breeding season and had a narrower peak compared to males (Fig. 2a and b; Table 4). The effect of wind speed was very similar in both males and females, with speeds above 8 m.s^− 1^ resulting in a significant increase in the size of reproductive aggregations (Fig. 2c and d; Table 4). Full moon decreased the size of reproductive aggregations in both males and females, whereas the size of reproductive aggregations peaked before and after the full moon phase (Fig. 2e and f; Table 4). The effects of time-dependent changes in discharge on the size of reproductive aggregations were significant but relatively small compared to the effects of other variables. Increasing discharge has different effects depending on hour in a day, with the size of reproductive aggregations decreasing in the evening and increasing in the morning. Males responded more quickly to discharge than females (Fig. 2g and h; Table 4). For both sexes, the size of reproductive aggregations peaked in the evening hours during the first half of breeding season, while hour in a day became less important during the second half of the breeding season (Fig. 3; Table 4).

**Table 3:** The estimated parametric coefficients and their significances (coefficients for water temperature, difference between air and water temperature, precipitation, and atmospheric pressure, respectively) in M1 and M2. The adjusted R^2^ of the male model M1 (N = 5569) was 0.56 and deviance explained 56.3%, while the female model M2 (N = 5537) was 0.488 and explained 49.3% of the deviance. Significant values of the explanatory variables are in bold

M1 - males	Estimate	Std. error	t value	p value
Intercept	-2.163	2.457	-0.881	0.379
Water temp (°C)	-0.003	0.0010	-0.299	0.765
Temp. diff. (Air-water)	0.084	0.006	14.322	**< 0.001**
Precipitation	0.283	0.102	2.768	**0.006**
Atm. pressure (kPa)	-0.060	0.026	-2.341	**0.019**
M2 - females				
Intercept	-28.828	2.023	-14.247	**< 0.001**
Water temp (°C)	0.122	0.010	12.801	**< 0.001**
Temp. diff. (Air-water)	0.063	0.005	12.804	**< 0.001**
Precipitation	0.371	0.085	4.385	**< 0.001**
Atm. pressure (kPa)	0.213	0.021	10.090	**< 0.001**

**Table 4:** Significance of smooth terms of variables (day in a season, interaction of day a season and hour in a day, wind speed, lunar phase and interaction of hour in a day ~ discharge) included in M1 and M2. The adjusted R^2^ of the male model M1 (N = 5569) was 0.56 and deviance explained 56.3%, while the female model M2 (N = 5537) was 0.488 and explained 49.3% of the deviance. Significant values of the explanatory variables are in bold

M1 - males	Edf	Ref.df	F	p value
Day in a season	8.758	8.946	36.162	**< 0.001**
Day ~ Hour in a day	12.806	18.000	23.357	**< 0.001**
Wind speed	6.190	7.340	5.833	**< 0.001**
Lunar phase	7.292	8.000	28.684	**< 0.001**
h in a day ~ discharge	7.570	8.483	11.018	**< 0.001**
M2 - females				
Day in a season	8.181	8.745	15.792	**< 0.001**
Day ~ Hour in a day	13.289	18.000	25.558	**< 0.001**
Wind speed	6.382	7.518	10.706	**< 0.001**
Lunar phase	7.551	8.000	46.223	**< 0.001**
Time of a day ~ discharge	6.908	8.037	7.168	**< 0.001**

## Discussion

Based on the robust dataset of 1702 individuals, seven years of observations, and a relatively large subsample of tracked individuals from the total population in the study site, we observed significant effects of water temperature, the difference between water and air temperature, precipitation, atmospheric pressure, day in a season and its interaction with hour in a day, wind speed, lunar phase, and discharge interacting with hour in a day. The model suggests evaluation of complex involvement of environmental cues by fish to time the size of their reproductive aggregations, as well as between-sex differences in response to environmental cues. To the authors’ knowledge, this is the first study to show such complex utilization of environmental cues to control reproductive aggregations in fish, highlighting the importance of this study for the phenological behaviour of freshwater fish.

Temperature is believed to be the main environmental cue for reproductive migration and the onset of reproduction in Cypriniformes in spring warming waters of temperate zones [60, 61]. Surprisingly, water temperature was only important for females in the GAM model. Males appeared to be driven by all other variables considered in the GAM model, but very strongly by difference between air and water temperature, suggesting that fish may be selecting warm water layers at days of strong migration [50]. The size of reproductive aggregations of males and females both responded strongly to variable integrating air and water temperatures, but this variable was much more important than temperature itself for males alone. Temperature usually plays a key role in the development of female gonads [24], and was therefore important in the female model. In general, the effects of temperature on reproductive aggregations of fish are consistent with research on fish migrations, since temperature was shown to affect various types of fish migration including feeding migrations [4], predator avoidance [62], and reproduction [63], and thus is regarded as a principal component of fish the decision process to migrate [62]. However, it seems that in asp breeding, temperature contribution to the size of reproductive aggregation is not so dominant, because in this case, it was also significantly influenced by many other environmental cues. Finally, it is important to note that, as in any non-manipulative, observational field study, we inevitably have some statistical relationship between seasonality and water temperature. The correlation between our implementation of the annual seasonality (as the relative position of a day since the start of a given breeding season) and water temperature is about 0.7. Surely, it is far from collinearity (and hence non-identifiability of the model), but it is possible that the separation between temperature and seasonal effects is not perfect and might contribute to relatively low and insignificant temperature effect for males.

Weather changes are also an essential component of environmental cues that can trigger migrations and reproductive onsets in many organisms, such as insects [64], fishes [22, 23], birds [65], and mammals [66]. This appears to be the case in the fish studied, asp, where precipitation increased the size of reproductive aggregations in both sexes and increase in atmospheric pressure decreased the size of reproductive aggregation in males and increased the size of reproductive aggregation in females. Thus, surprisingly, females were positively reacting to weather changes, while male response was slightly negative. The difference between the sexes may be attributed to their variable migration timings [38], which could explain different sex-specific needs in response to environmental cues.

In a species where females are more limited in maximum number of offspring compared to their counterparts, males undergo strong competition for females [36]. When reproduction occurs at a well-defined reproductive site, males attempt to maximize their chances by investing more time in reproduction, arriving earlier, and staying longer at the breeding grounds compared to females [29]. Therefore, the differences in sex-specific response to selected variables (water temperature, day in the season and hour in a day) by our models likely reflect the different migration strategies between males and females.

Another important variable that indicated migration was wind speed. Wind speed may be a proxy of weather changes, and it is also known to influence migration strength in other animal taxa, such as birds [67, 68] or insects [69]. In fishes, wind can indirectly trigger migration by affecting productivity through changes in water masses movement [70, 71] or changes in turbidity affecting migration [72]. In our models, the response to wind speed started approximately at speed of 8 m × s^− 1^, while there was very little or no response below that value for both sexes. Although we have no direct data to support this hypothesis, we assume that this threshold may be related to mixing of warmer water layers in the lower water column, which could trigger higher migration from staging grounds (located in standing water) to breeding grounds. Because the study site is located at the beginning of a canyon-shaped reservoir with steep shores that substantially block the wind, the lower wind speeds at flatter landscape may be enough to trigger movement of water masses and observed threshold value may be quite site-specific.

Sudden changes in water discharge due to hydropeaking may have profound effects on reproductive success [43, 73] and immediate reproductive effort of individual fish [40, 61]. On the other hand, an increase in water discharge usually has positive effects on upstream migrations [74], unless the change is extreme and occurs under temperature minima for swimming [40]. In our data, higher discharge did not have such a strong effect on migration, causing the size of reproductive aggregations to decrease at evening and nocturnal hours and increasing the size of reproductive aggregations during morning hours.

Hour in a day was an important variable affecting the size of reproductive aggregations of asp and interacting with day of a breeding season in predictable pattern. Overall, the largest reproductive aggregations occurs at night in asp reproduction, which may be advantageous due to intense egg predation by bleak (*Alburnus alburnus*) [20]. Predation is often the reason why reproductive migrations occur during the night and twilight hours [27, 28]. In our dataset, the possibility that largest reproductive aggregations occurs at night because of predation due to low light levels is also supported by the effects of lunar phase, which showed a trend of decreasing the size of reproductive aggregations during full moon), suggesting an optimization of the fish behaviour in the sense of “to see and not to be seen”. However, because our dataset included only moon phases and night-time cloud cover, we could not study the direct effect of light intensity upon the fish.

For fish that breed in the spring, the timing is essential for parental fitness to match fry food demands with peaks in zooplankton density [7, 8], which usually occurs in late spring in northern temperate zones [75]. Due to the asp egg development period after fertilization of about 20 days at common water temperature during egg development phase and further ten days of the yolk sack absorption [76], the offspring start foraging about 30 days after reproductive act of parents. Thus, the variability of the size of reproductive aggregations during breeding season and long duration of breeding for both individuals and the population may be adaptive to recruitment size in this species.

The male and female models explained about half of variability of acquired data, suggesting that future studies might consider additional environmental cues affecting the size of reproductive aggregations, as well internally controlled physiological processes [77]. One possibility is that density dependent effects contributed to variability of reproductive aggregations due to nonlinear (threshold-like) effects. Harsh competition among males could reduce the number of migrating individuals in breeding grounds due to limited space despite favourable environmental cues for migration. In addition, other cues such as synchronization among individuals through pheromone stimulation [78], variation in water turbidity [79] or social interactions [16] may also be relevant and contribute to migration. Because the performance of individual fish is not uniform throughout its reproductive lifetime but increases with age [29, 80], we may expect that changes in the age structure of contributing individuals across the years evaluated can also result in some variability in among years.

Although animal migrations have inspired hundreds of studies on their triggers, the current availability of big data from detailed tracking of animal movement [81] to publicly available weather data [47] enable researchers to test combinations of interaction between environmental cues and migration trends that were previously hard to be achieved. This may lead to increased knowledge on migratory triggers that can also be applied to conservation measures. Another important issue is the unexplained part of the variability of reproductive aggregations that still accounts for about 50%. Future research focusing on migrating individuals may shed light on the contribution of social interactions to this variability.

## Data Availability

The datasets analysed during the current study are available from the corresponding author upon request.

## References

[1] Alerstam T, Hedenström A, Åkesson S (2003). Long-distance migration: evolution and determinants. Oikos.

[2] Chapman BB, Hulthén K, Brodersen J, Nilsson PA, Skov C, Hansson LA (2012). Partial migration in fishes: causes and consequences. J Fish Biol.

[3] Candino M, Donadio E, Pauli JN (2022). Phenological drivers of ungulate migration in South America: characterizing the movement and seasonal habitat use of guanacos. Mov Ecol.

[4] Mehner T. Diel vertical migration of freshwater fishes - proximate triggers, ultimate causes and research perspectives. Freshw. Biol. 2012;57:1342–59.

[5] Sajdlová Z, Frouzová J, Draštík V, Jůza T, Peterka J, Prchalová M (2018). Are diel vertical migrations of european perch (*Perca fluviatilis* L.) early juveniles under direct control of light intensity? Evidence from a large field experiment. Freshw Biol.

[6] Childress ES, Mcintyre PB (2015). Multiple nutrient subsidy pathways from a spawning migration of iteroparous fish. Freshw Biol.

[7] Reglero P, Ortega A, Balbín R, Abascal FJ, Medina A, Blanco E et al. Atlantic bluefin tuna spawn at suboptimal temperatures for their offspring. Proceedings Biol Sci. 2018;285:20171405. 10.1098/rspb.2017.1405 PMC5784186 29321292

[8] Cushing DH (1990). Plankton Production and Year-class Strength in Fish populations: an update of the Match/Mismatch hypothesis. Adv Mar Biol.

[9] Lerche-Jørgensen M, Korner-Nievergelt F, Tøttrup AP, Willemoes M, Thorup K (2018). Early returning long-distance migrant males do pay a survival cost. Ecol Evol.

[10] Møller AP (1994). Phenotype-dependent arrival time and its consequences in a migratory bird. Behav Ecol Sociobiol.

[11] Hulthén K, Chapman BB, Nilsson PA, Hansson L, Skov C, Brodersen J et al. Timing and synchrony of migration in a freshwater fish: consequences for survival. J. Anim. Ecol. 2022;91(10):2103–12. 10.1111/1365-2656.13790 PMC9805062 35899786

[12] Acácio M, Catry I, Soriano-Redondo A, Silva JP, Atkinson PW, Franco AMA (2022). Timing is critical: consequences of asynchronous migration for the performance and destination of a long-distance migrant. Mov Ecol.

[13] Burnside RJ, Salliss D, Collar NJ, Dolman PM (2021). Birds use individually consistent temperature cues to time their migration departure. Proc Natl Acad Sci U S A.

[14] Winkler DW, Jørgensen C, Both C, Houston AI, McNamara JM, Levey DJ (2014). Cues, strategies, and outcomes: how migrating vertebrates track environmental change. Mov Ecol.

[15] Lucas MC, Baras E. Migration of Freshwater Fishes. Lucas MC, Baras E, Thom TJ, Duncan A, Slavk O, editors. Oxford, UK; 2001.

[16] Berdahl A, Westley PAH, Quinn TP (2017). Social interactions shape the timing of spawning migrations in an anadromous fish. Anim Behav.

[17] Tibblin P, Forsman A, Borger T, Larsson P (2016). Causes and consequences of repeatability, flexibility and individual fine-tuning of migratory timing in pike. J Anim Ecol.

[18] Sims DW, Wearmouth VJ, Genner MJ, Southward AJ, Hawkins SJ (2004). Low-temperature-driven early spawning migration of a temperate marine fish. J Anim Ecol.

[19] Perkin JS, Gido KB, Cooper AR, Turner TF, Osborne MJ, Johnson ER (2015). Fragmentation and dewatering transform Great Plains stream fish communities. Ecol Monogr.

[20] Šmejkal M, Souza AT, Blabolil P, Bartoň D, Sajdlová Z, Vejřík L (2018). Nocturnal spawning as a way to avoid egg exposure to diurnal predators. Sci Rep.

[21] Forsythe PS, Scribner KT, Crossman JA, Ragavendran A, Baker EA, Davis C (2012). Environmental and lunar cues are predictive of the timing of river entry and spawning-site arrival in lake sturgeon Acipenser fulvescens. J Fish Biol.

[22] Katselis G, Koukou K, Dimitriou E, Koutsikopoulos C (2007). Short-term seaward fish migration in the Messolonghi–Etoliko lagoons (western greek coast) in relation to climatic variables and the lunar cycle. Estuar Coast Shelf Sci.

[23] de Magalhães Lopes J, Alves CBM, Peressin A, Pompeu PS (2018). Influence of rainfall, hydrological fluctuations, and lunar phase on spawning migration timing of the neotropical fish *Prochilodus costatus*. Hydrobiologia.

[24] Migaud H, Davie A, Taylor JF. Current knowledge on the photoneuroendocrine regulation of reproduction in temperate fish species. J. Fish Biol. 2010;76(1):27–68. 10.1111/j.1095-8649.2009.02500.x 20738699

[25] Souza AT, Ilarri MI, Timóteo S, Marques JC, Martins I (2018). Assessing the effects of temperature and salinity oscillations on a key mesopredator fish from european coastal systems. Sci Total Environ.

[26] Pankhurst NW, Porter MJR (2003). Cold and dark or warm and light: variations on the theme of environmental control of reproduction. Fish Physiol Biochem.

[27] Robertson DR. The ecology of fishes on coral reefs. In: Sale P, editor. Ecol Fishes Coral Reefs. 1st editio. Academic Press LTD; 1993. p. 356–86.

[28] Finlay RW, Poole R, French AS, Phillips KP, Kaufmann J, Doogan A (2020). Spawning-related movements in a salmonid appear timed to reduce exposure to visually oriented predators. Anim Behav.

[29] Šmejkal M, Bartoň D, Brabec M, Sajdlová Z, Souza AT, Moraes KR (2021). Climbing up the ladder: male reproductive behaviour changes with age in a long-lived fish. Behav Ecol Sociobiol.

[30] Morbey YE (2003). Pair formation, pre-spawning waiting, and protandry in kokanee, *Oncorhynchus nerka*. Behav Ecol Sociobiol.

[31] Šmejkal M, Ricard D, Vejřík L, Mrkvička T, Vebrová L, Baran R (2017). Seasonal and daily protandry in a cyprinid fish. Sci Rep.

[32] Trail PW, Adams ES (1989). Active mate choice at cock-of-the-rock leks: tactics of sampling and comparison. Behav Ecol Sociobiol.

[33] Apollonio M, De Cena F, Bongi P, Ciuti S. Female preference and predation risk models can explain the maintenance of a fallow deer (*Dama dama*) lek and its “handy” location. PLoS One. 2014;9(3):e89852. 10.1371/journal.pone.0089852 PMC3943860 24599036

[34] Rotics S, Kaatz M, Turjeman S, Zurell D, Wikelski M, Sapir N (2018). Early arrival at breeding grounds: causes, costs and a trade-off with overwintering latitude. J Anim Ecol.

[35] Pärt T (2001). Experimental evidence of environmental effects on age-specific reproductive success: the importance of resource quality. Proc R Soc B Biol Sci.

[36] Morbey YE, Ydenberg RC (2001). Protandrous arrival timing to breeding areas: a review. Ecol Lett.

[37] Kokko H, Gunnarsson TG, Morrell LJ, Gill J. a. Why do female migratory birds arrive later than males? J Anim Ecol. 2006;75:1293–303. 10.1111/j.1365-2656.2006.01151.x 17032361

[38] Šmejkal M, Bartoň D, Brabec M, Sajdlová Z, Souza AT, Moraes KR (2022). Behaviour affects capture probability by active sampling gear in a cyprinid fish. Fish Res.

[39] Briedis M, Bauer S, Adamík P, Alves JA, Costa JS, Emmenegger T et al. A full annual perspective on sex-biased migration timing in long-distance migratory birds. Proc. R. Soc. B. 2019;286:20182821. 10.1098/rspb.2018.2821 PMC6408886 30963841

[40] Bartoň D, Brabec M, Sajdlová Z, Souza AT, Duras J, Kortan D (2022). Hydropeaking causes spatial shifts in a reproducing rheophilic fish. Sci Total Environ Elsevier.

[41] Schiemer F, Waidbacher H. Strategies for conservation of a danubian fish fauna. In: Boon PJ, Calow P, Petts GJ, editors. River Conserv Manag. 1st ed. John Wiley & Sons; 1992. pp. 363–82.

[42] Balon EK (1975). Reproductive guilds of fishes: a proposal and definition. J Fish Res Board Canada.

[43] Bartoň D, Blabolil P, Sajdlová Z, Vejřík L, Souza AT, Kubečka J (2021). Effects of hydropeaking on the attached eggs of a rheophilic cyprinid species. Ecohydrology.

[44] Miranda LE (2005). Refining Boat Electrofishing Equipment to improve consistency and reduce harm to Fish. North Am J Fish Manag.

[45] Šmejkal M, Blabolil P, Bartoň D, Duras J, Vejřík L, Sajdlová Z (2019). Sex-specific probability of PIT-tag retention in a cyprinid fish. Fish Res.

[46] Castro-Santos T, Haro A, Walk S. A passive integrated transponder (PIT) tag system for monitoring fishways. Fish Res. 1996;28:253–61.

[47] Rienecker MM, Suarez MJ, Gelaro R, Todling R, Bacmeister J, Liu E (2011). MERRA: NASA’s Modern-Era Retrospective Analysis for Research and Applications. J Clim.

[48] NASA. NASA POWER | Prediction Of Worldwide Energy Resources. 2020.

[49] NASA. Global Modeling and Assimilation Office (GMAO). Greenbelt, MD, USA, Goddard Earth Sciences Data. and Information Services Center (GES DISC); 2022.

[50] Garner P, Clough S, Griffiths SW, Deans D, Ibbotson A (1998). Use of shallow marginal habitat by *Phoxinus phoxinus*: a trade-off between temperature and food?. J Fish Biol.

[51] Lazaridis E. lunar: Lunar Phase & Distance, Seasons and Other Environmental Factors. 2014.

[52] Chrysafi A, Jepsen N, del Villar-Guerra D, Larsen MH, Skov C (2021). Effects of passive integrated transponder tags on short-term feeding patterns in european perch (*Perca fluviatilis*). J Fish Biol.

[53] Burnett NJ, Stamplecoskie KM, Thiem JD, Cooke SJ (2013). Comparison of detection efficiency among three sizes of half-duplex passive integrated transponders using manual tracking and fixed antenna arrays. North Am J Fish Manag.

[54] Connolly PJ, Jezorek IG, Martens KD, Prentice EF (2008). Measuring the performance of two stationary interrogation systems for detecting downstream and upstream movement of PIT-tagged salmonids. North Am J Fish Manag Wiley.

[55] Hastie TJ. Generalized additive models. New York Routledge; 2017.

[56] Wood SN. Generalized additive models: An introduction with R, second edition. Gen. Addit. Model. An Introd. with R, Second Ed. Chapman & Hall; 2017.

[57] Boor C (2001). A practical guide to Splines - revised Edition.

[58] Hastie T, Tibshirani R, Varying-Coefficient, Models (1993). J R Stat Soc Ser B.

[59] R Core Team RD. R Development Core Team, R: a language and environment for statistical computing. R A Lang.Environ. Estat. Comput. 2022.

[60] Hladík M, Kubečka J (2003). Fish migration between a temperate reservoir and its main tributary. Hydrobiologia.

[61] King J, Cambray JA, Impson ND (1998). Linked effects of dam-released floods and water temperature on spawning of the Clanwilliam yellowfish *Barbus capensis*. Hydrobiologia.

[62] Brönmark C, Skov C, Brodersen J, Nilsson PA, Hansson L-A (2008). Seasonal migration determined by a trade-off between predator avoidance and growth. PLoS ONE.

[63] Jansen T, Gislason H (2011). Temperature affects the timing of spawning and migration of North Sea mackerel. Cont Shelf Res.

[64] Srygley RB, Dudley R, Oliveira EG, Aizprúa R, Pelaez NZ, Riveros AJ (2010). El Niño and dry season rainfall influence hostplant phenology and an annual butterfly migration from neotropical wet to dry forests. Glob Chang Biol.

[65] Haest B, Hüppop O, Bairlein F (2018). The influence of weather on avian spring migration phenology: what, where and when?. Glob Chang Biol.

[66] Kauffman MJ, Aikens EO, Esmaeili S, Kaczensky P, Middleton A, Monteith KL (2021). Causes, Consequences, and conservation of Ungulate Migration. Annu Rev Ecol Evol Syst Annual Reviews.

[67] Erni B, Liechti F, Underhill LG, Bruderer B (2002). Wind and rain govern the intensity of nocturnal bird migration in central Europe - A log-linear regression analysis. Ardea -Wageningen.

[68] Liechti F (2006). Birds: Blowin’ by the wind?. J Ornithol.

[69] Knight SM, Pitman GM, Flockhart DTT, Norris DR. Radio-tracking reveals how wind and temperature influence the pace of daytime insect migration.Biol Lett. 2019;15. 10.1098/rsbl.2019.0327 PMC6684972 31266418

[70] Rodríguez-Basalo A, Punz A, Ceballos-Roa E, Jordà G, Manuel González-Irusta J, Massutí E (2022). Fisheries-based approach to disentangle mackerel (*Scomber scombrus*) migration in the Cantabrian Sea. Fish Oceanogr.

[71] Jarić I, Říha M, Souza AT, Rabaneda-Bueno R, Děd V, Gjelland K (2022). Influence of internal seiche dynamics on vertical movement of fish. Freshw Biol.

[72] Sudo R, Okamura A, Fukuda N, Miller MJ, Tsukamoto K (2017). Environmental factors affecting the onset of spawning migrations of japanese eels (*Anguilla japonica*) in Mikawa Bay Japan. Environ Biol Fishes.

[73] Casas-Mulet R, Saltveit SJ, Alfredsen K (2015). The survival of Atlantic Salmon ( * Salmo salar * ) Eggs during dewatering in a river subjected to Hydropeaking. River Res Appl.

[74] Taylor MK, Cooke SJ (2012). Meta-analyses of the effects of river flow on fish movement and activity. Environ Rev.

[75] Straile D (2015). Zooplankton biomass dynamics in oligotrophic versus eutrophic conditions: a test of the PEG model. Freshw Biol.

[76] Targoñska K, Zarski D, Kucharczyk D (2008). A review of the artificial reproduction of asp, *Aspius aspius* (L.), and nase, *Chondrostoma nasus* (L). Arch Pol Fish.

[77] Woods T, Kaz A, Giam X. Phenology in freshwaters: a review and recommendations for future research. Ecography (Cop). 2022;2022:e05564.

[78] Sorensen PW, Wisenden BD. Fish pheromones and related cues. Fish pheromones relat. cues. Wiley Blackwell; 2015.

[79] Childs AR, Cowley PD, Næsje TF, Booth AJ, Potts WM, Thorstad EB (2008). Do environmental factors influence the movement of estuarine fish? A case study using acoustic telemetry. Estuar Coast Shelf Sci Academic Press.

[80] Rebke M, Coulson T, Becker PH, Vaupel JW (2010). Reproductive improvement and senescence in a long-lived bird. Proc Natl Acad Sci U S A.

[81] Nathan R, Monk CT, Arlinghaus R, Adam T, Alós J, Assaf M et al. Big-data approaches lead to an increased understanding of the ecology of animal movement. Science (80-). American Association for the Advancement of Science; 2022;375. 10.1126/science.abg1780 35175823

[82] QGIS Development Team. QGIS Geographic Information System. Open Source Geospatial Found. Proj. 2016.

